# Supplementary material for: Plasma Cytokines/Chemokines as Predictive Biomarkers for Lymphedema in Breast Cancer Patients
Source: Cancers (Basel). 2023 Jan 21;15(3):676. doi: 10.3390/cancers15030676 (PMC9913278; doi:10.3390/cancers15030676)
Supplement: Supplementary file 1 [file cancers-15-00676-s001.zip › cancers-2145200-supplementary.pdf]

Full inclusion and exclusion criteria for recruiting study subjects

Inclusion criteria:

1) Participants must be at least 18 years of age

2) Participants must be clinically diagnosed with breast cancer
3) Patients must plan to undergo treatment with surgery and radiation therapy at MDACC

4) Clinical stage N2-N3; or clinical stage N1 with an intention to treat with axillary lymph node dissection and regional nodal radiation

5) Ambulatory and possessing all four limbs

6) No prior radiation therapy targeted to lymph nodes

7) Fluency in English or Spanish.

Exclusion Criteria:

1) Participants with a known or suspected allergy to iodine

2) Participants who are breastfeeding, pregnant or trying to become pregnant

3) Severe underlying chronic illness or disease (other than breast cancer)
4) Participants not capable of keeping moderately still for the imaging portion of the study session (~1 hour for imaging)

We will review medical and family history as we analyze data collected at each of the six study visits.

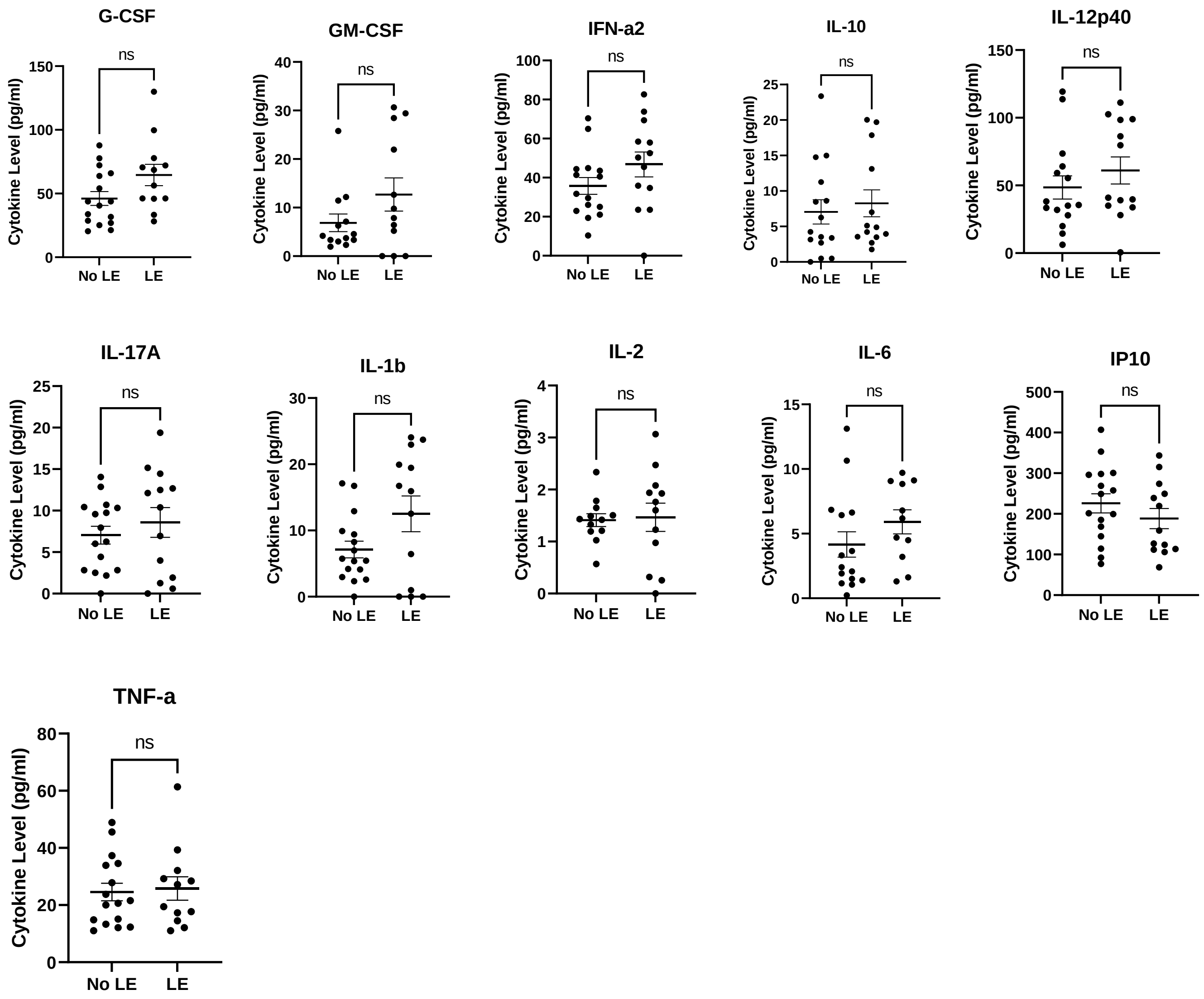

Supplemental Figure S1. Statistically non-significant plasma cytokines/chemokines at 12 months post-RT in subjects with clinical BCRL at 12 months post-RT.

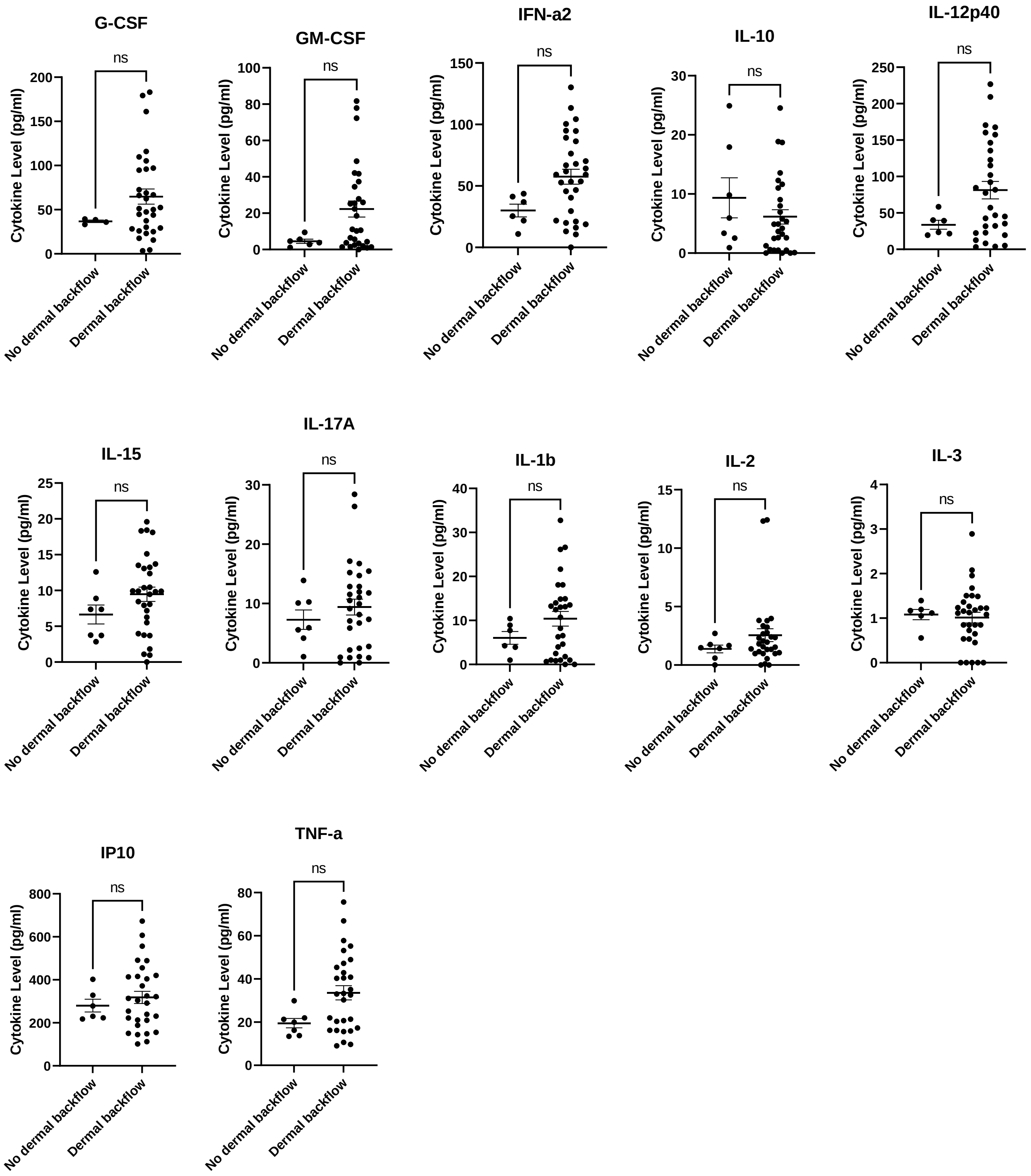

Supplemental Figure S2. Pre -ALND cytokine/chemokine levels were statistically non-significant in subjects displaying dermal backflow at 12 months post-RT.

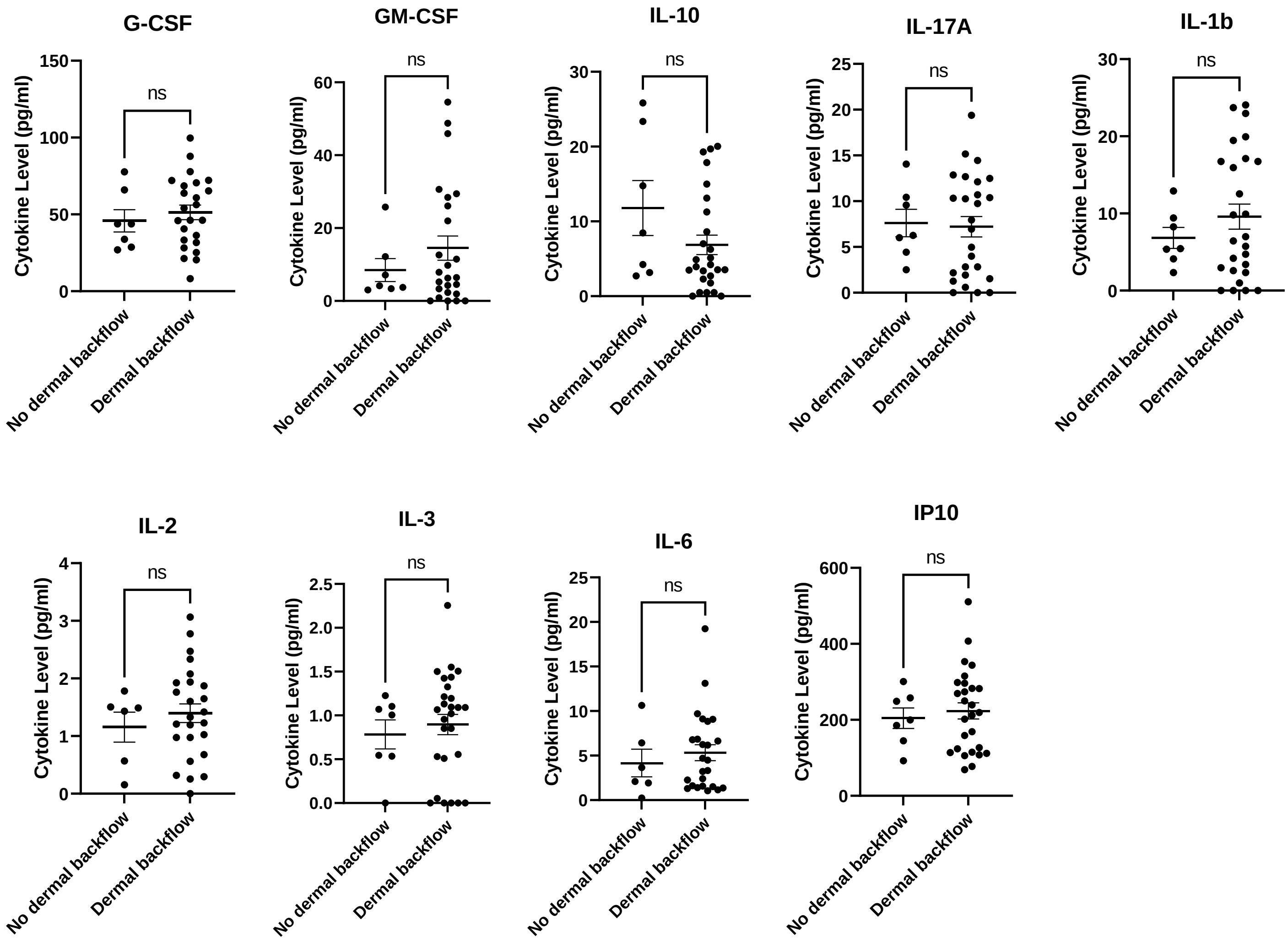

Supplemental Figure S3. Plasma cytokine/chemokine levels at 12 months post-RT were non-significant in subjects with dermal backflow at 12 months post-RT.

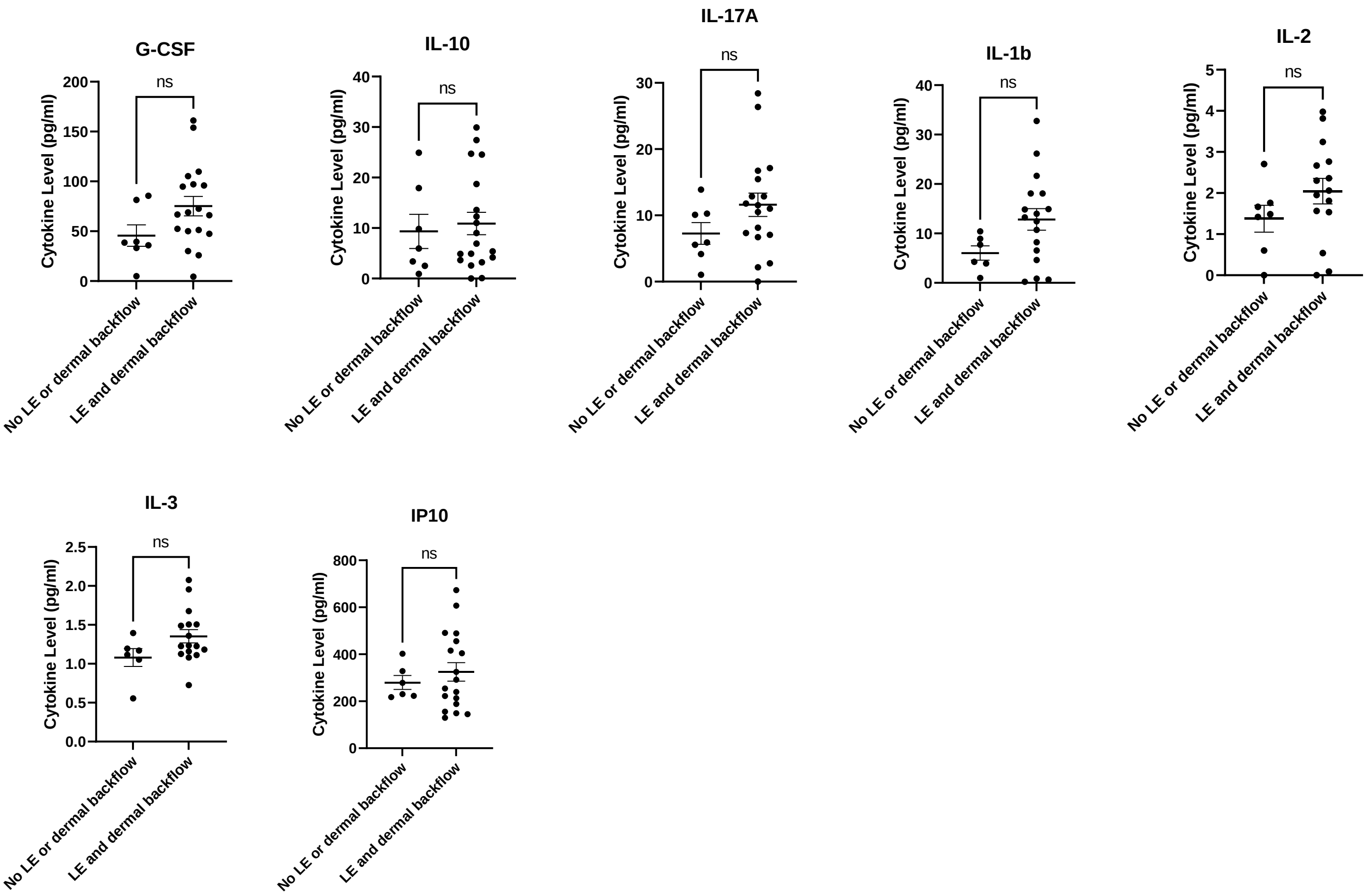

Supplemental Figure S4. Non-significant plasma cytokine/chemokine levels at pre-ALND in subjects with both clinical BCRL and dermal backflow at 12 months post-RT compared to those without clinical BCRL or dermal backflow.

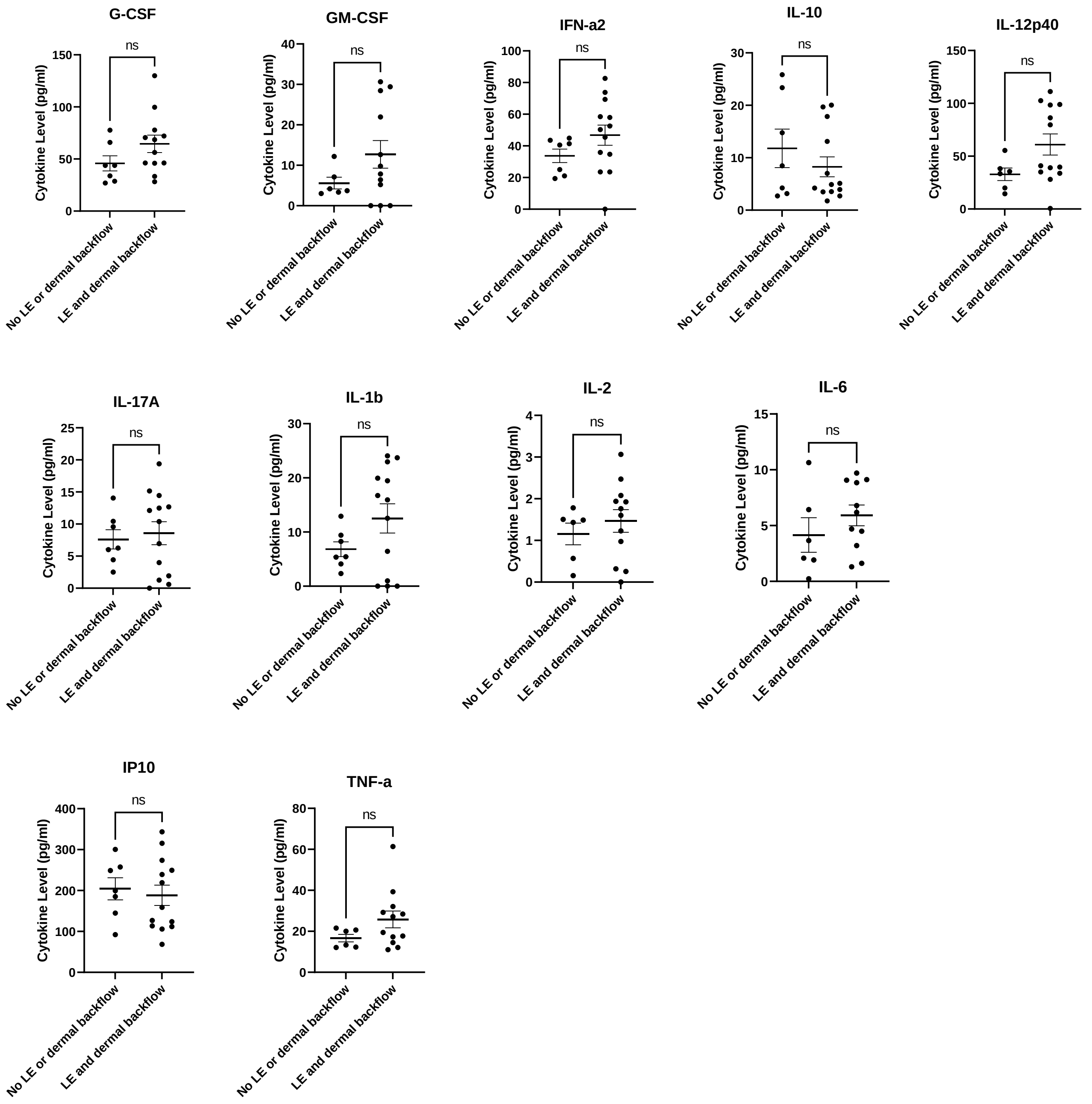

Supplemental Figure S5. Plasma cytokine/chemokine levels at 12 months post-RT were statistically non-significant in those with both clinical LE and dermal backflow at 12 months post-RT compared to those with neither clinical BCRL nor dermal backflow.

STROBE Checklist/Table

**Item and Number**	**Responses to recommendations or Locations within Manuscript**	**Page Number(s) or Location**
1) Title and abstract	a) Predictive biomarkers for lymphedema in breast cancer patients<br>b) Abstract cohesively communicates what was done and found	a) Page 1<br>b) Page 1 - Abstract
**Introduction**		
2) Background/rationale		Introduction
3) Objectives	Identify breast cancer patients at highest risk for BCRL before clinical diagnosis	Introduction - last paragraph
**Methods**		
4) Study Design	Longitudinal, observational study over a year's time	Manuscript section 2.1
5) Setting	MD Anderson Cancer Center, Houston, TX and University of Texas Health Sciences Center, Houston, TX	Manuscript section 2.1
6) Participants	a) A total of 80 consented participants but only 40 were used in analysis<br>b) Full inclusion and exclusion of subject recruitment are located in supplementary data	a) Manuscript section 2.1
7) Variables	a) Plasma cytokine/chemokine levels<br>b) %RVC values<br>c) %EVD values	a) Manuscript section 2.6<br>b) Manuscript section 2.3<br>c) Manuscript section 2.4
8) Data sources/measurement	a) NIRF- LI imaging device and session were used for determination of dermal backflow<br>b) Perometric score measurements	a) Manuscript section 2.2 and 2.7<br>b) Manuscript section 2.3
9) Bias	MBA, JCR, and EMS independently assessed data collected, to avoid any bias in interpretation	
10) Study size	A total of 80 consented participants but only 40 were used in analysis	Manuscript section 2.1
11) Quantitative variables	a) RVC scores $\geq$ 5% were diagnosed with clinical LE<br>b) P-values < 0.05 were deemed significant	a) Manuscript section 2.3<br>b) Manuscript section 2.7
12) Statistical methods	Significance was calculated using Mann-Whitney non-parametric t-test on GraphPad Prism and Pearson's correlation values were calculated from Excel	Manuscript section 2.7
**Results**		
13) Participants	A total of 80 subjects consented for study but only 40 subjects had sufficient data for analysis	Manuscript section 2.1 and discussion- last paragraph
14) Descriptive data	Table 1 lists the demographics of all 40 subjects	
15) Outcome data		
16) Main results	Figures 3-8 and Supplemental figures 1-5 graphically depicts	

	significance and standard error of the mean for each cytokine	
17) Other analyses	Table 2 lists correlation values for all cytokines for each comparison group	
**Discussion**		
18) Key results	a) Plasma cytokine/chemokine levels can predict who is most at risk in developing BCRL<br>b) There is an ongoing cycle of inflammation associated with BCRL	Manuscript sections 4 and 5
19) Limitations	Small sample size and tracking of compression use	Discussion – last paragraph
20) Interpretation	Discussion and Conclusions	Manuscript sections 4 and 5
21) Generalizability	Conclusion	Manuscript section 5
Other information		
22) Funding	NIH and CPRIT	Funding and Acknowledgement
